# Antioxidant Effects of Curcumin Gel in Experimental Induced Diabetes and Periodontitis in Rats

**DOI:** 10.1155/2022/7278064

**Published:** 2022-05-10

**Authors:** Chenar Anwar Mohammad, Khadeeja Mohammed Ali, Aram Mohammed Sha, Sarhang Sarwat Gul

**Affiliations:** ^1^Periodontics Department, College of Dentistry, Hawler Medical University, Erbil, Iraq; ^2^Periodontics Department, College of Dentistry, Sulaimani University, Sulaimani, Iraq; ^3^Smart Health Tower, Sulaimani, Iraq

## Abstract

This study aimed to evaluate the effect of curcumin gel on antioxidant marker level in experimental induced diabetes and periodontitis (EDP) in rats. Adult Wistar rats were randomized into five groups (20 each): (1) EDP treated with scaling and root planing (SRP) + curcumin gel (CU), (2) EDP treated with CU, (3) EDP treated with SRP, (4) EDP without treatment, and (5) systemically healthy and without ligature (control). Each group was subdivided equally into 4 subgroups of 5 rats. Diabetes was induced by intraperitoneal injection of streptozotocin (STZ), and periodontitis was induced by a ligature. Blood samples were collected by cardiac puncture at 0, 7, 14, and 21 days to assess oxidative stress of malondialdehyde (MDA) and antioxidant enzymes of glutathione peroxidase (GPx), catalase (CAT), and suproxidase dismutase (SOD) levels. The results showed a significant increase in serum MDA and antioxidant enzyme levels in the untreated EDP group compared to the control group (*p* < 0.05). The adjuvant use of CU to SRP resulted in a significant reduction of MDA and CAT levels as compared to the SRP group (*p* < 0.05); however, significant reduction of GPX and SOD levels can be found only at day 7. It can be concluded that the decreased level of antioxidant enzymes can be construed as a result of decreased oxidative stress by curcumin therapy.

## 1. Introduction

Diabetes mellitus is a group of metabolic diseases characterized by hyperglycemia resulting from defects in insulin secretion, a defect in insulin action, or a combination of both. In turn, the deficiency causes chronic hyperglycemia with disruptions in carbohydrate, fat, and protein metabolism [1]. Periodontitis is an inflammatory disease set off by gram-negative bacteria residing in the subgingival biofilm, leading to the destruction of tooth supporting structures [2]. Considerable evidence has indicated a relationship between periodontitis and noncommunicable diseases such as diabetes, heart diseases, and chronic kidney disease, and periodontitis has been considered the sixth complication of diabetes after retinopathy, nephropathy, neuropathy, cardiovascular disease, and peripheral vascular disease [3, 4].

In periodontitis, periodontal pathogens and their products activate the host defense mechanisms and induce the formation of reactive oxygen species (ROS) or free radicals which are released by the activated PMNs and cause oxidative killing of bacteria in biofilms [5]. Persistent exposure to ROS leads to a wide spectrum of pathologic reactions in host tissue [6] and contributes to the pathogenesis of various inflammatory diseases, through harmful oxidative reactions which range from periodontal diseases to systemic diseases such as diabetes mellitus and cardiovascular diseases [5, 7].

In healthy individuals, protection against the harmful effects of ROS on cells is obtained by maintaining a balance between oxidants and antioxidants. In a balanced cell state, ROS are produced as a normal product of cellular metabolism, and the level of ROS can be stabilized by an antioxidant defense system, including antioxidant enzymes [8]. Antioxidant enzymes protect tissues against oxidative injury by scavenging free oxygen radicals generated by various metabolic processes, modulating the extent of the inflammatory response. The most important intracellular enzymes which protect cells and tissues from oxygen-derived free radicals are glutathione peroxidase (GPx), catalase (CAT), and suproxidase dismutase (SOD) [5]. While, in a state of cellular imbalance, the levels of oxidants outweigh the levels of antioxidants, and if a cell is exposed to more reactive oxygen compounds than it can instantly degrade, it is under oxidative stress [8].

The most commonly used criterion of oxidative stress is based on the determination of the peroxidation products of lipids, particularly polyunsaturated fatty acid residues of phospholipids, which are susceptible to attack by ROS [9]. Malondialdehyde (MDA) is one of the final products of the peroxidation of polyunsaturated fatty acids in cells. An increase in free radicals causes the overproduction of MDA. Therefore, the MDA level is commonly known as a marker of oxidative stress and peroxidative tissue injury [10].

The fundamental treatment of periodontitis is to reduce the load of subgingival pathogenic bacteria by scaling and root planing (SRP) or surgical procedures [11]. Although SRP represents the standard procedure for periodontal treatment, it may fail to remove some pathogenic organisms, mainly due to the difficulty of accessing as in furcation regions or deep pockets. Additionally, some virulent bacterial strains are thought to penetrate the epithelial lining of the periodontal pocket, appear inaccessible to instrumentation, and potentially lead to reinfection of the pocket [12]. Consequently, in conjunction with SRP, a rationale for the use of antibiotics emerges [13]. Adjunctive antibiotics are used in periodontitis either as locally or systemically delivered agents. However, the development of resistance against antibiotics and the side effects of the drugs implicate the search for alternatives. Thus, the search continues for newer and safer therapeutic agents to overcome these limitations. Phytochemicals isolated from plants are considered good alternatives to synthetic chemicals.

Curcumin, a yellow pigment from Curcuma longa, is a major component of turmeric. It possesses antiinflammatory, antioxidant [14], antimicrobial properties [15], along with its hepatoprotective, immunostimulant, antiseptic, antimutagenic, and many more properties. In recent years, the use of curcumin extracts has gained popularity, and it has been used as adjunct to SRP to treat periodontitis or repair bone defects [16].

Meanwhile, there is no information related to the effect of local delivery of prepared curcumin gel (alone or as adjunct with SRP) on oxidative stress marker and antioxidant potential in experimental induced diabetes and periodontitis in rats. Therefore, the aim of the present study is to determine the effect of locally delivered prepared curcumin gel in the correction of antioxidant system disorders in the experimental induced diabetes and periodontitis in rats through measurement of serum oxidative stress marker (MDA) and antioxidant enzyme activity levels (GPx, CAT, and SOD) after different time intervals of therapy.

## 2. Materials and Methods

### 2.1. Rats and Housing

For this study, 100 male Wister Albino rats, aged 8-10 weeks and weighing 250-280 g, were selected. The experiments were carried out following the principles of laboratory animal care (NIH publication 85-23, 1985). The study was approved by the Ethics Committee for Animal Research, College of Dentistry, Hawler Medical University, Erbil, Iraq. All rats were allowed to adapt to the housing conditions for one week prior to the commencement of the study. The animals were kept and housed on a 12-hour light/dark cycle at 20 ± 5 °C and 20%-30% humidity in a temperature-controlled room, in plastic cages identified by their group types and periods, and they were fed the laboratory rat food and tap water ad libitum before, during, and after therapy. Five rats were housed in each cage for the study.

### 2.2. Induction of Diabetes in Rats

Diabetes was induced by a single intraperitoneal injection of freshly prepared streptozotocin (STZ) (45 mg/kg-body weight (b.w.)) in citrate buffer 4.5 pH. After 72 hrs, the level of blood glucose from the tail vein was determined, which was found to be >16.7 mmol/L; this indicated that the rats had been induced with diabetes mellitus [17]. Blood glucose was measured by snipping the tails of fasting animals using a standard glucometer. Body weight and blood glucose were also measured weekly during the entire study.

### 2.3. Induction of Ligature Induced Periodontitis in Rats

Experimental periodontitis (EP) was established in 80 rats. General anesthesia was achieved through intramuscular injection with a solution of ketamine 10% and xylazine 2% (2 : 1), 0.12 ml/100 g body weight. Anesthesia was installed in 4-5 min after administration. The animals were placed on a proper operating table, which allowed for open-mouth maintenance of the rats to facilitate access to their teeth. Ligatures in “8” with 4/0 nonresorbable sterile silk thread were placed around the cervical region of the mandibular incisor region. This ligature acted as a gingival irritant for 14 days and promoted the accumulation of plaque and subsequently the development of periodontal disease [18]. After placing ligatures, the animals were kept in the same conditions, 5 rats in each cage. Rats were observed for 21 days. Ligature control was performed daily, and the animals were checked to ensure proper nutrition [18].

### 2.4. Experimental Design

The experimental animals were randomly selected for inclusion in groups: (1) intact healthy animals, without ligature induced periodontitis (control group *n* = 20), and (2) animals with experimentally induced diabetes and periodontitis, without treatment, named EDP (*n* = 20), and the remaining 60 rats with experimentally induced diabetes and periodontitis, which received treatments, were included in the experimental diabetes periodontitis treated group and were separated randomly into the following 3 groups (each group *n* = 20):

- First group: EDP rats treated by SRP followed by local application of prepared curcumin gel (12.5 *μ*g/ml) for 7 days, called SCU group
- Second group: EDP rats treated by local application of prepared Cu gel (12.5 *μ*g/ml) alone for 7 days, called CU group
- Third group: EDP treated by scaling and root planing alone, called SRP group

Each treated group was divided randomly into 4 subgroups of 5 rats, and a blood sample was collected from each subgroup at the baseline before therapy (0 day) and after 7, 14, and 21 days of therapy.

### 2.5. Diabetes Periodontitis Therapy

For the diabetes and periodontitis treatment groups, ligatures were removed on day 14, and the treatment was performed one day after ligature removal for all 3 treatment groups. In the first SCU and third SRP treated groups, mandibular incisors were subjected to scaling and root planing with manual #1–2 mini-five curettes, using 10 distal–mesial traction movements in the labial and lingual aspects, and cervical–incisal traction movements were used for the interproximal areas [19]. Then, the gel was subgingivally delivered, immediately after SRP, into the mandibular incisor using a plastic syringe with a blunt needle, and this was repeated once daily for 7 days. For the second treated group, CU, the rats also received curcumin gel alone for 7 days.

### 2.6. Curcumin Gel Preparation

For both CU and SCU groups, the rats were treated with12.5 *μ*g/ml curcumin gel [20] that consisted of 95% curcumin (Bulk Supplements Pure Curcumin 95% Natural Turmeric Extract Powder), potassium sorbate (Analitik Kimya Ve Lab. Cih. San. Tic. Ltd. Sti. Istanbul, Turkey), propylene glycol (Pharmaco-Aaper, Bengaluru, India), metalose 90SH 10000 (Shin-Etsu Chemical Co., Chiyoda, Japan), and purified water. The gel was formulated by Awamedica Company, Hawler, Kurdistan Region, Iraq.

### 2.7. Blood Sample Collection

The animals were euthanized, and then blood was collected by cardiac puncture. The blood sample was transferred into a gel tube, centrifuged at 3,000 r.p.m. for 10 minutes, and dissociated to serum. Then, the acquired serum samples were collected and stored at -80 °C for subsequent analyses of MDA, GPx, CAT, and SOD activity levels. The blood samples were collected from control and untreated EDP rats at 0 (meaning after 7 days of animal housing in control group and immediately after ligature induced periodontitis removal in the EDP), 7, 14, and 21 days and from three treated groups at 0 day (after ligature induced periodontitis removal and before therapy) and after different time intervals of treatment (7, 14, and 21 days).

### 2.8. Determination of Oxidative Stress Marker and Antioxidant Enzymes

GPx, CAT, SOD, and MDA were estimated in rat serum, based on enzyme-linked immunosorbent assays (ELISA) kits according to manufacturer's instruction. Rat ELISA kit (SunLog Biotech Co., LTD, China) was used for determination of MDA, rat catalase ELISA kit-NP_001734-BioMySource was used for determination of catalase, rat glutathione perioxidase1 (Gpx1) E:ISA kit-BioMySource was used for determination of glutathione perioxidase, and rat superoxidase dismutase (SOD) ELISA Kit-BioMySource was used for determination of superoxidase dismutase.

### 2.9. Statistical Analysis

The data were presented as mean ± standard deviation. The dataset was statistically analyzed using the SPSS software package (version 22; SPSS Inc., Chicago, IL, USA). The data were all normally distributed. The Dunnett *t* test was used to compare between control and different groups in relation to 0, 7, 14, and 21 days, with one-way ANOVA test (*F*-test) to compare between control, EDP, SCU, CU, and SRP groups at 0 day and after 7, 14, and 21 days of therapy. The Tukey HSD test was also used to compare between EDP and different treated groups at different time intervals of 0, 7, 14, and 21 days, with one-way ANOVA (*F*-test) to compare between EDP, SCU, CU, and SRP at 0, 7, 14, and 21 days. A paired *t*-test was also used to compare between 0 day and different time intervals for each group.

## 3. Results

Table 1 shows the mean value ± standard deviation of MDA, GPX, CAT, SODs, glucose levels, and body weight levels during all duration periods of 0, 7, 14, and 21 days, with paired *t* test comparison between 0 day (baseline) and different time intervals of 7, 14, and 21 days for each group. Significant differences were found between 0 days and different time intervals after therapy in all three treated groups, except for between day 0 and 7 days for GPXs in SCU group and between day 0 and 14 days for CAT in the CU group. For SOD, significant differences were found between day 0 and all time intervals in the SRP and CU groups (except for between 0 and 21 days in the CU group) and nonsignificant differences between day 0 and 7 days and 0 and 21 days in the SCU group.

For blood glucose levels, nonsignificant differences were found between 0 day and different time intervals of 7, 14, and 21 days in the control, SCU, and CU groups, with significant differences between 0 day and three different time intervals of therapy in the EDP group and between 0 and 7 and 0 and 14 days in the SRP group. For the body weight, significant differences were seen between 0 day and three different time intervals of 7, 14, and 21 days in the control, EDP, SCU, CU, and SRP groups.

### 3.1. Serum MDA

Table 2 shows that the serum MDA levels had significantly increased in the EDP group when compared to the control group in all studied periods of 0, 7, 14, and 21 days and there were significant differences between the control group and the three treated groups in all studied periods, with the exception of SCU at 21 days of therapy. Table 2 also shows that the local administration of CU alone and/or as adjunct with SRP and SRP resulted in a significant reduction of MDA after 7, 14, and 21 days of therapy as compared to the EDP group, with significant differences between the EDP group and the three treated groups. The comparison between the SCU and CU groups showed significant differences after 7, 14, and 21 days of therapy.

### 3.2. Serum GPX

Table 3 shows that the serum GPX levels in the EDP group had significantly increased when compared to the control group in all studied periods of 0, 7, 14, and 21 days, with no significant differences found between each treated group and control group after 7 days (with the exception of the SCU group) and 14 and 21 days of therapy. The local administration of CU alone and/or as adjunct with SRP and SRP resulted in gradual reduction of GPX level after different days of therapy as compared to EDP group, but with nonsignificant differences after 7 and 14 in SCU group and all studied periods of therapy in CU and SRP groups. Finally, the comparison between the SCU and CU groups showed nonsignificant differences after 14 and 21 days of therapy.

### 3.3. Serum CAT

Table 4 shows a significant increase in serum CAT level in EDP group as compared to control in all studied periods, with significant differences between control and the three treated groups after 7 and 14 days of therapy (with the exception of SRP at 14 days) and nonsignificant differences after 21 days of therapy in three treated groups as compared to control. Table 4 also shows that the local administration of CU alone and/or as adjunct with SRP resulted in nonsignificant reduction of CAT as compared to EDP after 7 days of therapy and significant reduction after 14 and 21 days of therapy, with no significant differences between SCU group and CU groups after 7, 14, and 21 days of therapy, while SRP resulted in a significant reduction of CAT after 7, 14, and 21 days of therapy as compared to EDP.

### 3.4. Serum SODs

Table 5 shows significant increase in serum SODs level in EDP group as compared to control in all studied periods, with nonsignificant differences between the control group and the three treated groups after 7 (with the exception of SCU), 14, and 21 days of therapy. Table 5 also shows that the local administration of CU as adjunct with SRP in SCU group resulted in nonsignificant reduction of SODs as compared to EDP after 7 days of therapy and significant reduction after 14 and 21 days of therapy, while the local administration of CU alone or SRP resulted in a significant reduction of SODs levels after 7, 14, and 21 days of therapy. The comparison between SCU group and CU group showed no significant differences after 14 and 21 days of therapy.

### 3.5. Glucose Level and Body Weight

Table 6 shows significant increase in blood glucose levels in treated EDP as compared to control group in all studied periods of 0, 7, 14, and 21 days. The comparison between control and the three treated groups showed significant differences after 7, 14, and 21 days of therapy. Table 6 also shows that the local administration of CU alone and/or as adjunct with SRP and SRP resulted in slight significant reduction of glucose levels after 7, 14, and 21 days of therapy compared to untreated EDP, but remaining significantly more than control, with nonsignificant differences found between each of two treated groups after 7 (with exception of CU and SRP groups), 14, and 21 days after therapy.

Table 7 shows significant reduction in the mean value of body weight in EDP without treatment at different time intervals of 0, 7, 14, and 21 days as compared to control. The local administration of CU alone and/or as adjunct with SRP and SRP resulted in slight nonsignificant elevation in the mean value of body weight as compared to untreated EDP group after 7, 14, and 21days of therapy, but with nonsignificant differences between each of the two groups, and between the four groups (EDP, SCU, CU, and SRP).

## 4. Discussion

The consideration that oxidative stress plays a central role in periodontal disease and SRP presents a limitation in certain cases has provided a rationale for using pharmacological agents adjunctively to mechanical therapy as antioxidants. Hence, curcumin was used in the current study over other products because it is purely natural, safe, and nontoxic. It possesses a broad spectrum of biological activities including antimicrobial, antiinflammatory, antioxidant properties [15, 21] and antidiabetic activity, and several studies showed that curcumin was effective in treating periodontal disease [22, 23].

MDA was evaluated as a biomarker of oxidative stress since MDA is well-established as a lipid peroxidation product to evaluate oxidative stress, and it is the most investigated lipid peroxidation product in periodontitis [24].

The present finding revealed that the serum level of MDA was significantly increased in the experimental induced diabetes and periodontitis untreated group (EDP) as compared to the control group for all duration periods of 0, 7, 14, and 21 days. This increase may have been due to the fact that periodontitis was in active state and the peripheral blood neutrophils exhibited a hyperactive phenotype in terms of the production of ROS, which may have diffused into the blood stream [7, 25]. This result was supported by a study on experimentally induced periodontitis in hyperglycemic Wistar rats [14] and demonstrated a significant elevation of MDA serum level in the untreated EDP group as compared to the control group. In the same line, a study [26] reported higher MDA levels in the periodontal tissue of patients with type 2 diabetes than in controls. Furthermore, crocin as another herbal ingredient of saffron that has been examined in relation to MDA, SOD, CAT, and GPx levels. Similar to the current study, increase levels of MDA, SOD, and CAT in experimentally induced periodontitis have been reported as compared to control group [20]. Whereas the GPx level was decreased, this can been explained by the fact that different herbal product was used as adjunct to SRP. Similar findings of the protective role of crocin on the cardiac and kidney tissues have been reported when crocin used as adjunct to SRP in experimentally induced periodontitis as compared to the control group [27, 28], indicating that crocin can be used as a protective agent in periodontitis induced inflammation and oxidative damage. These findings encourage examining the protective role of CU beyond periodontal tissue such as cardiac and kidney tissues in experimentally induced periodontitis.

The data of the current study also showed that the treatment with curcumin gel as adjuvant to SRP/and or curcumin alone resulted in a significant reduction of serum MDA levels when compared with untreated EDP group after different time intervals of 7, 14, and 21 days of therapy, but with a more significant reduction of MDA levels in the SCU group than CU alone. This effect can be attributed to the antioxidant action of curcumin and the fact that SRP is the gold standard treatment and curcumin can be used as adjunct to SRP. Our results corroborate the results of the previous studies which reported that curcumin minimizes the increase in MDA in experimentally induced periodontitis in diabetic rat models [14]. This finding is also in agreement with a study demonstrating that curcumin exerts an antioxidant property by reducing several oxidative stress markers such as MDA [29].

Studies of enzymatic antioxidants in relation to diabetes mellitus patients with periodontal disease are limited, and the results are contradictory. Some studies reported higher antioxidant activity [30–32], while other studies found lower antioxidant activity [14, 33, 34]. In the present study, SOD, CAT, and GPx were selected, since they are the most important antioxidant enzymes for removing ROS and inhibiting the toxic effects of oxidant molecules on tissues and cells [35].

The results of the present study showed a significant elevation of the mean values of SOD, GPx, and CAT in the serum of the untreated EDP group as compared to the control group in all the study time period. This elevation may be explained due to several mechanisms: First, the disturbance in the endogenous antioxidant defense system, maybe due to the overproduction of lipid peroxidation products at inflammatory sites, can be related to a greater degree of oxidative stress production [30]; second, *Porphyromonas gingivalis* induces ROS production in the gingival epithelial cells, and to counteract the damaging effects of ROS, the host cell stimulates a number of antioxidant mechanisms [36, 37] which include the production of catalase, superoxide dismutase, and peroxidase [38]; and third, the increase of antioxidant levels in rats with experimentally induced diabetes and periodontitis may indicate the continuous activation of the immune response during disease to afford biological protection against excessive generated lipid peroxidation products [39].

Our results are consistent with a study conducted to determine total antioxidant capacity and SOD levels in the serum of diabetes patients and healthy control with and without periodontal disease. It reported that diabetics with periodontitis exhibited higher SOD levels as compared with the control group [32]. Additionally, our results are consistent with a study that investigated SOD activity levels in type-2 diabetes and systemically healthy individuals with periodontitis [31] and demonstrated that diabetics with periodontitis had the highest SOD levels. Furthermore, another study reported that the elevation of SOD activity level in inflamed gingiva during periodontal disease may be due to the increased O2 generation by polymorph nuclear leukocytes invading the disease situation. The increase in O2 production may have led to the occurrence of oxidative stress, which in turn produced an increased need for SOD production to establish the ROS-antioxidant balance to protect the tissue [40]. Liu et al. [41] reported the elevation of SOD levels in patients with periodontitis. They explained that the increased stimulation of SOD production protects against biological superoxide generation in periodontal inflammation. Another study evaluated the gene expression of GPx in the gingival tissue in poorly and well-controlled type 2 diabetics with periodontitis, demonstrating that GPx was overexpressed in diabetics with periodontitis as compared to controls [42].

In contrast to our result, clinical studies reported that antioxidant levels of SOD, CAT, and GPx were decreased significantly in diabetic patients with periodontitis as compared to controls [33, 34]. Also, an experimental study by Li et al. (2018) reported that the serum activity of SOD in the experimentally induced diabetes and periodontitis group was decreased when compared to the control group [43]. This discrepancy in the results may be attributed to the differences in the severity of periodontal disease and time-dependent changes in the enzyme activity.

Regarding the local treatment, the present study showed that the curcumin gel when administrated as an adjuvant to SRP and or alone resulted in the gradual continuous reduction of GPx, SOD, and CAT activity levels as compared to untreated EDP after different time intervals of 7, 14, and 21 days of therapy. However, the mean value of GPX and SOD remained significantly more than the control only after 7 days of therapy in the SCU group, and the mean value of CAT remained significantly more than the control after 7 and 14 days in both SCU and CU groups. In the same line, a study reported that the oral administration of curcumin for streptozotocin-induced diabetic rats resulted in a decreased SOD expression. This low level of SOD activity after treatment could reflect diminished oxidative stress [44].

The antioxidant activity of curcumin is attributed to its chemical structure, which contains phenolic hydroxyl and methoxy-groups, which are responsible for radical scavenging activity, and a central methylenic moiety capable of H-atom donation and breaking chain oxidation reaction [45].

However, in the present study, the mean activity level of GPx and SOD remained significantly higher than the control after 7 days of therapy in the SCU group only, with nonsignificant differences from the control after 14 and 21 days. Also, the mean activity level of CAT remained significantly higher than in the control for all time intervals of 7, 14, and 21 days of therapy in SCU group and after 7 and 14 days in CU group. This result provides direct evidence that curcumin exhibits antioxidant effect on periodontal tissue caused by oxidative stress induced by both diabetes and ligature placement.

Several studies reported that curcumin possesses the capacity to scavenge free radicals, reduce the generation of ROS, and act as strong inhibitor of lipid peroxidation and advanced glycation end products [46, 47]. Therefore, based on this information and the present data for SOD, GPXs, and CAT levels in rats with experimentally induced diabetes and periodontitis which were treated with curcumin as an adjuvant to SRP and/or alone, it can be proposed that curcumin reduces the oxidative damage in periodontitis by inhibiting the generation of ROS. The reduction of serum antioxidant levels may thus represent one of the possible explanations for the antioxidant effect of curcumin against periodontal disease.

To the contrary, a study demonstrated that the oral administration of curcumin as a monotherapy for the treatment of ligature-induced periodontitis in diabetic rats for 10 weeks duration resulted in a significant elevation of glutathione and catalase levels [14]. This discrepancy in the results may be attributed to the differences in the periodontal treatment method or to the difference in the route of curcumin administration or possibly to the duration and dose of curcumin, since the antioxidant activity of curcumin depends on the dose of curcumin and the duration of treatment [47].

Regarding the effect of local periodontal treatment used in this study, the resolution of periodontal inflammation with SRP resulted in a significant reduction of MDA and antioxidant enzyme levels after 7, 14, and 21 days of therapy as compared to 0 day before therapy, due to removal of noxious stimuli by SRP, which led to less generation of ROS [48].

In addition to oxidative stress, our results showed that EDP at baseline (day 0) and untreated EDP group were characterized by hyperglycemia and weight loss as compared to the control group. These results were consistent with other studies [43]; both experimental diabetes and periodontitis models were successfully established in rats and characterized by hyperglycemia, weight loss, and oxidative stress. The hyperglycemia in STZ-induced diabetic rats could have resulted from the destruction of pancreatic *β*-cell by the action of STZ injection [49], and the decreased body weight may have occurred as a consequence of hyperglycemia and compromised mastication resulting from ligature placement around the tooth [50].

However, the local treatment by curcumin delivery (in the SCU group and CU group) or by nonsurgical periodontal therapy resulted in a slight significant reduction of glucose levels after treatment compared to untreated EDP or baseline (day 0), but the results remained more significant than for the control in all treated groups and with a nonsignificant elevation of body weight.

The slight significant continuous reduction in blood glucose level may possibly have resulted from resolution of serum inflammatory factors as well as a possible reduction of glucolipid metabolism and insulin resistance by mechanical periodontal therapy [51] or may have been due to the effect of curcumin in minimizing the increase in blood glucose level in STZ-induced diabetes rat models [44].

The use of curcumin for health reasons has mainly been in traditional and folk medicine. Therefore, it is difficult to pinpoint the exact recommended standard dose for this herb. Different dosages of curcumin have been used by researchers: 30 and 100 (mg/kg b.w.) [52], 2% of curcumin gel in the treatment of experimental periodontitis [53],10 mg of synthetic curcumin gel for the treatment of periodontitis [54], and 75 (mg/kg b.w.) in an experimental study [14]. The selection of 12.5 *μ*g/ml curcumin gel in the current study was based on an experimental study [15, 16].

In the present study, the local curcumin delivery approach was selected over systemic approaches, since animal studies reported low systemic bioavailability following oral administration of curcumin, which results in poor absorption and rapid metabolism [55]. Furthermore, curcumin has shown better retention within the periodontal pocket because of its bioadhesive property [56].

## 5. Conclusion

The significant reduction in the serum level of oxidative stress marker (MDA) and antioxidant enzymes (SOD, CAT, and GPx) in rats that received local treatment with curcumin provides direct evidence that local administration of curcumin has an antioxidant effect of ameliorating periodontitis induced by ligature in diabetic rats.

## Figures and Tables

**Table 1:** The mean value of MDA, GPX, CAT, SOD, glucose, and body weight in all groups with comparison between baseline 0 day and different time intervals (7, 14, and 21 days) for each group.

		Mean ± SD				
Parameters	Time	Control	EDP	SCU	CU	SRP
MDA (ng/ml)	0	56.2 ± 1.64	216.6 ± 2.7	216.4 ± 2.4	216.6 ± 2.6	217 ± 2
	7	56 ± 1.58	216.5 ± 2.69	91.92 ± 1.88∗∗	100.8 ± 0.84∗∗	105.4 ± 1.51∗∗
	14	55.8 ± 1.64	216.1 ± 2.43	77.68 ± 1.76∗∗	83.42 ± 3.21∗∗	88.12 ± 1.6∗∗
	21	55.6 ± 1.81	216 ± 2.23	57.4 ± 1.14∗∗	64.42 ± 2.7∗∗	71.44 ± 0.74∗∗

	0	558.1 ± 110.3	1632 ± 244.4	1632 ± 244.3	1631 ± 244.2	1631 ± 244.1
GPX (pg/ml)	7	557.3 ± 110.1∗∗	1632 ± 244.2	1466 ± 114.3	654.8 ± 177.2∗∗	643.2 ± 187.9∗∗
	14	557.1 ± 110.1∗∗	1630 ± 244.2∗	550 ± 66.83∗∗	416 ± 39.75∗∗	393.7 ± 12∗∗
	21	557.2 ± 110.1	1628.94 ± 244.28	426 ± 87.89∗∗	406.3 ± 42.02∗∗	384.8 ± 9.17∗∗

CAT (pg/ml)	0	783.8 ± 63.56	2021 ± 18.59	2020 ± 18.26	2020 ± 18.57	2020 ± 18.57
	7	783.5 ± 63.61∗	2020 ± 18.58	1828 ± 47.86∗∗	1794 ± 253.2	1036 ± 200.4∗∗
	14	783.6 ± 63.38	2020 ± 18.20	1348 ± 159.9∗∗	1282 ± 317.5∗∗	944 ± 191.1∗∗
	21	783.4 ± 63.50	2020 ± 18.30	809.4 ± 22.4∗∗	788.3 ± 22.78∗∗	726.6 ± 58.47∗∗

SOD (ng/ml)	0	0.97 ± 0.29	5.34 ± 1.94	5.42 ± 1.78	5.44 ± 1.75	5.44 ± 1.65
	7	0.97 ± 0.34	5.28 ± 1.93	3.76 ± 1.18	1.39 ± 0.33∗∗	1.06 ± 0.07∗∗
	14	0.97 ± 0.29	5.22 ± 1.95	1.02 ± 0.2∗∗	1 ± 0.25∗∗	0.87 ± 0.27∗∗
	21	0.97 ± 0.36	5.26 ± 1.97∗∗	0.87 ± 0.13	0.89 ± 0.18	0.85 ± 0.008∗

GLU (pg/dl)	0	114 ± 1.07	383.1 ± 1.98	382.7 ± 1.75	382.6 ± 1.72	382.8 ± 1.69
	7	114.1 ± 1.97	388.9 ± 0.93∗∗	383.1 ± 1.98∗∗	382 ± 1.71	379.7 ± 1.97∗∗
	14	114.1 ± 1.96	397.8 ± 5.06∗∗	383.1 ± 1.98∗∗	384.8 ± 7.23	379 ± 1.24∗
	21	114.2 ± 1.11	406.6 ± 2.86∗	382 ± 2.3∗	382.1 ± 3.37	378.3 ± 2.02

BW (gr)	0	275.4 ± 3.37	268.8 ± 5.66	268.4 ± 5.58	268.3 ± 5.47	268.3 ± 5.54
	7	278.9 ± 3.5∗∗	262.4 ± 7.17∗∗	263 ± 6.51∗∗	267.2 ± 7.45∗∗	267.6 ± 7.13∗∗
	14	282.2 ± 3.17∗∗	257.1 ± 5.44∗∗	257.7 ± 5.31∗∗	258 ± 5.19∗	258.1 ± 2.21∗
	21	289.7 ± 2.49∗∗	251.7 ± 2.65∗∗	253.1 ± 2.75∗∗	254.3 ± 2.81∗∗	256.1 ± 1.31∗∗

EPD: experimental diabetes and periodontitis; SCU: scaling and curcumin; CU: curcumin; SRP: scaling and root planing; MDA: malondialdehyde; GPX: glutathione peroxidase; CAT: catalase; GLU: glucose, BW: body weight; ∗∗highly significant; ∗significant.

**Table 2:** The comparison between the mean value of MDA in the control and different groups using Dunnett *t* test and Tukey HSD test to compare MDA between EDP and three treated groups. The comparison among different groups using one-way ANOVA.

		0 day		7 days		14 days		21days	
Variable	Groups	Mean ± SD	*P*	Mean ± SD	*P*	Mean ± SD	*P*	Mean ± SD	*P* value

MDA (ng/ml)	C	56.2 ± 1.64	≤0.001	56 ± 1.58	≤0.001	55.8 ± 1.64	≤0.001	55.6 ± 1.82	≤0.001
	EDP	216.6 ± 2.7		216.5 ± 2.69		216.08 ± 2.24		216 ± 2.24	
	C	56.2 ± 1.64	≤0.001	56 ± 1.58	≤0.001	55.8 ± 1.64	≤0.001	55.6 ± 1.82	0.38
	SCU	216.4 ± 2.41		91.92 ± 1.89		77.68 ± 1.76		57.4 ± 1.14	
	C	56.2 ± 1.64	≤0.001	56 ± 1.58	≤0.001	55.8 ± 1.64	≤0.001	55.6 ± 1.82	≤0.001
	CU	216.6 ± 2.61		100.8 ± 0.84		83.42 ± 3.21		64.42 ± 2.71	
	C	56.2 ± 1.64	≤0.001	56 ± 1.58	≤0.001	55.8 ± 1.64	≤0.001	55.6 ± 1.82	≤0.001
	SRP	217 ± 2.00		105.4 ± 1.52		88.12 ± 1.61		71.44 ± 0.75	

*P* value		≤0.001†		≤0.001†		≤0.001†		≤0.001†	

MDA (ng/ml)	EDP	216.6 ± 2.7	0.99	216.5 ± 2.69	≤0.001	216.08 ± 2.24	≤0.001	216 ± 2.24	≤0.001
	SCU	216.4 ± 2.41		91.92 ± 1.89		77.68 ± 1.76		57.4 ± 1.14	
	EDP	216.6 ± 2.7	1.000	216.5 ± 2.69	≤0.001	216.08 ± 2.24	≤0.001	216 ± 2.24	≤0.001
	CU	216.6 ± 2.61		100.8 ± 0.84		83.42 ± 3.21		64.42 ± 2.71	
	EDP	216.6 ± 2.7	0.99	216.5 ± 2.69	≤0.001	216.08 ± 2.24	≤0.001	216 ± 2.24	≤0.001
	SRP	217 ± 2		105.4 ± 1.52		88.12 ± 1.61		71.44 ± 0.75	
	SCU	216.4 ± 2.41	0.99	91.92 ± 1.89	≤0.001	77.68 ± 1.76	0.006	57.4 ± 1.14	≤0.001
	CU	216.6 ± 2.61		100.8 ± 0.84		83.42 ± 3.21		64.42 ± 2.71	
	SCU	216.4 ± 2.41	0.97	91.92 ± 1.89	≤0.001	77.68 ± 1.76	≤0.001	57.4 ± 1.14	≤0.001
	SRP	217 ± 2		105.4 ± 1.52		88.12 ± 1.61		71.44 ± 0.75	
	CU	216.6 ± 2.61	0.99	100.8 ± 0.84	0.006	83.42 ± 3.21	0.02	64.42 ± 2.71	≤0.001
	SRP	217 ± 2		105.4 ± 1.52		88.12 ± 1.61		71.44 ± 0.75	

*P* value		0.983†		≤0.001†		≤0.001†		≤0.001†	

MDA: malondialdehyde; C: control; EPD: experimental diabetes and periodontitis; SCU: scaling and curcumin; CU: curcumin; SRP: scaling and root planing; †one-way ANOVA; *P*: probability <0.05 is significant; *P* > 0.05 is nonsignificant; *P* < 0.01 is highly significant.

**Table 3:** The comparison between the mean value of GPXs in control and different groups using Dunnett *t* test and Tukey HSD test to compare GPXs between EDP and three treated groups. The comparison among different groups using one-way ANOVA.

Variable	Groups	0 day		7 days		14 days		21 days	
		Mean ± SD	*P*	Mean ± SD	*P*	Mean ± SD	*P*	Mean ± SD	*P*
GPX (pg/ml)	C	558.15 ± 110.32	≤0.001	557.32 ± 110.14	≤0.001	557.21 ± 110.1	≤0.001	558.02 ± 110.47	≤0.001
	EDP	1632.19 ± 244.3		1631.6 ± 244.22		1630.08 ± 244.2		1628.94 ± 244.2	
	C	558.15 ± 110.32	≤0.001	557.32 ± 110.14	≤0.001	557.21 ± 110.1	1	558.02 ± 110.47	1
	SCU	1631.68 ± 244.3		1465.93 ± 114.6		550.02 ± 66.83		425.97 ± 87.89	
	C	558.15 ± 110.32	≤0.001	557.32 ± 110.14	0.78	557.21 ± 110.1	0.25	558.02 ± 110.47	1
	CU	1631.3 ± 244.24		654.77 ± 177.23		415.96 ± 39.75		406.26 ± 42.02	
	C	558.15 ± 110.32	≤0.001	557.32 ± 110.14	0.85	557.21 ± 110.1	0.1	558.02 ± 110.47	1
	SRP	1631.12 ± 244.1		643.17 ± 187.93		393.7 ± 12		384.82 ± 9.17	

*P* value		≤0.001†		≤0.001†		≤0.001†		≤0.001†	

GPX (pg/ml)	EDP	1632.19 ± 244.3	1	1631.6 ± 244.22	0.51	1630.08 ± 244.2	≤0.001	1628.94 ± 244.2	≤0.001
	SCU	1631.68 ± 244.3		1465.93 ± 114.6		550.02 ± 66.83		425.97 ± 87.89	
	EDP	1632.19 ± 244.3	1	1631.6 ± 244.22	≤0.001	1630.08 ± 244.2	≤0.001	1628.94 ± 244.2	≤0.001
	CU	1631.3 ± 244.24		654.77 ± 177.23		415.96 ± 39.75		406.26 ± 42.02	
	EDP	1632.19 ± 244.3	1	1631.6 ± 244.22	≤0.001	1630.08 ± 244.2	≤0.001	1628.94 ± 244.2	≤0.001
	SRP	1631.12 ± 244.1		643.17 ± 187.93		393.7 ± 12		384.82 ± 9.17	
	SCU	1631.68 ± 244.3	1	1465.93 ± 114.6	≤0.001	550.02 ± 66.83	0.3	425.97 ± 87.89	1
	CU	1631.3 ± 244.24		654.77 ± 177.23		415.96 ± 39.75		406.26 ± 42.02	
	SCU	1631.68 ± 244.3	1	1465.93 ± 114.6	≤0.001	550.02 ± 66.83	0.2	425.97 ± 87.89	1
	SRP	1631.12 ± 244.1		643.17 ± 187.93		393.7 ± 12		384.82 ± 9.17	
	CU	1631.3 ± 244.24	1	654.77 ± 177.23	1	415.96 ± 39.75	0.9	406.26 ± 42.02	1
	SRP	1631.12 ± 244.1		643.17 ± 187.93		393.7 ± 12		384.82 ± 9.17	

*P* value		1.000 †		≤0.001†		≤0.001†		≤0.001†	

GPX: glutathione peroxidase; C: control; EPD: experimental diabetes and periodontitis; SCU: scaling and curcumin; CU: curcumin; SRP: scaling and root planing; †one-way ANOVA; *P*: probability <0.05 is significant; *P* > 0.05 is nonsignificant.

**Table 4:** The comparison between the mean level of CAT in control and different groups using Dunnett *t* test and Tukey HSD test to compare CAT between untreated EDP and three treated groups. The comparison among different groups using one-way ANOVA.

Variable	Groups	0 day		7 days		14 days		21 days	
		Mean ± SD	*P*	Mean ± SD	*P*	Mean ± SD	*P*	Mean ± SD	*P*
CAT (pg/ml)	C	783.77 ± 63.56	≤0.001	783.53 ± 63.61	≤0.001	783.55 ± 63.38	≤0.001	783.36 ± 63.5	≤0.001
	EDP	2020.88 ± 18.59		2020.44 ± 18.58		2019.76 ± 18.2		2019.64 ± 18.3	
	C	783.77 ± 63.56	≤0.001	783.53 ± 63.61	≤0.001	783.55 ± 63.38	≤0.001	783.36 ± 63.5	0.72
	SCU	2020.49 ± 18.26		1828.19 ± 47.86		1347.95 ± 159.9		809.35 ± 22.4	
	C	783.77 ± 63.56	≤0.001	783.53 ± 63.61	≤0.001	783.55 ± 63.38	≤0.001	783.36 ± 63.5	0.99
	CU	2019.93 ± 18.57		1793.75 ± 253.2		1281.67 ± 317.5		788.31 ± 22.78	
	C	783.77 ± 63.56	≤0.001	783.53 ± 63.61	0.04	783.55 ± 63.38	0.45	783.36 ± 63.5	0.13
	SRP	2019.93 ± 18.57		1035.95 ± 231.4		943.97 ± 191.09		726.61 ± 58.47	

*P* value		≤0.001 (HS)†		≤0.001 (HS)†		≤0.001 (HS)†		≤0.001 (HS)†	

CAT (pg/ml)	EDP	2020.88 ± 18.59	1	2020.44 ± 18.58	0.28	2019.76 ± 18.2	≤0.001	2019.64 ± 18.3	≤0.001
	SCU	2020.49 ± 18.26		1828.19 ± 47.86		1347.95 ± 159.9		809.35 ± 22.4	
	EDP	2020.88 ± 18.59	1	2020.44 ± 18.58	0.16	2019.76 ± 18.2	≤0.001	2019.64 ± 18.3	≤0.001
	CU	2019.93 ± 18.57		1793.75 ± 253.2		1281.67 ± 317.5		788.31 ± 22.78	
	EDP	2020.88 ± 18.59	1	2020.44 ± 18.58	≤0.001	2019.76 ± 18.2	≤0.001	2019.64 ± 18.3	≤0.001
	SRP	2019.93 ± 18.57		1035.95 ± 231.4		943.97 ± 191.09		726.61 ± 58.47	
	SCU	2020.49 ± 18.26	1	1828.19 ± 47.86	0.98	1347.95 ± 159.9	0.95	809.35 ± 22.4	0.77
	CU	2019.93 ± 18.57		1793.75 ± 253.2		1281.67 ± 317.5		788.31 ± 22.78	
	SCU	2020.49 ± 18.26	1	1828.19 ± 47.86	≤0.001	1347.95 ± 159.9	0.028	809.35 ± 22.4	0.008
	SRP	2019.93 ± 18.57		1035.95 ± 231.4		943.97 ± 191.09		726.61 ± 58.47	
	CU	2019.93 ± 18.57	1	1793.75 ± 253.2	≤0.001	1281.67 ± 317.5	0.075	788.31 ± 22.78	0.05
	SRP	2019.93 ± 18.57		1035.95 ± 231.4		943.97 ± 191.09		726.61 ± 58.47	

*P* value		1.000†		≤0.001†		≤0.001†		≤0.001†	

CAT: catalase; C: control; EPD: untreated experimental diabetes and periodontitis; SCU: scaling and curcumin; CU: curcumin; SRP: scaling and root planing; †one-way ANOVA; *P*: probability <0.05 is significant; *P* > 0.05 is nonsignificant.

**Table 5:** The comparison between the mean value of SODs in control and different groups using Dunnett *t* test and Tukey HSD test to compare glucose level between untreated EDP and three treated groups. The comparison among different groups using one-way ANOVA.

Variable	Groups	0 day		7 days		14 days		21 days	
		Mean ± SD	*P*	Mean ± SD	*P*	Mean ± SD	*P*	Mean ± SD	*P*
SOD (ng/ml)	C	0.97 ± 0.3	0.001	0.97 ± 0.34	≤0.001	0.97 ± 0.3	≤0.001	0.97 ± 0.37	≤0.001
	EDP	5.34 ± 1.94		5.28 ± 1.94		5.22 ± 1.96		5.26 ± 1.98	
	C	0.97 ± 0.3	0.001	0.97 ± 0.34	0.001	0.97 ± 0.3	1	0.97 ± 0.37	0.999
	SCU	5.42 ± 1.79		3.76 ± 1.19		1.02 ± 0.21		0.87 ± 0.13	
	C	0.97 ± 0.3	0.001	0.97 ± 0.34	0.91	0.97 ± 0.3	1	0.97 ± 0.37	1.000
	CU	5.44 ± 1.76		1.39 ± 0.34		1 ± 0.26		0.89 ± 0.18	
	C	0.97 ± 0.3	0.001	0.97 ± 0.34	1	0.97 ± 0.3	0.99	0.97 ± 0.37	0.999
	SRP	5.44 ± 1.65		1.07 ± 0.08		0.87 ± 0.28		0.85 ± 0.01	

*P* value		≤0.001†		≤0.001†		≤0.001†		≤0.001†	

SOD (ng/ml)	EDP	5.34 ± 1.94	1	5.28 ± 1.94	0.19	5.22 ± 1.96	≤0.001	5.26 ± 1.98	≤0.001
	SCU	5.42 ± 1.79		3.76 ± 1.19		1.02 ± 0.21		0.87 ± 0.13	
	EDP	5.34 ± 1.94	1	5.28 ± 1.94	≤0.001	5.22 ± 1.96	≤0.001	5.26 ± 1.98	≤0.001
	CU	5.44 ± 1.76		1.39 ± 0.34		1 ± 0.26		0.89 ± 0.18	
	EDP	5.34 ± 1.94	1	5.28 ± 1.94	≤0.001	5.22 ± 1.96	≤0.001	5.26 ± 1.98	≤0.001
	SRP	5.44 ± 1.65		1.07 ± 0.08		0.87 ± 0.28		0.85 ± 0.01	
	SCU	*SCU* *vs* *CU*	1	3.76 ± 1.19	0.023	1.02 ± 0.21	1	0.87 ± 0.13	1
	CU	5.44 ± 1.76		1.39 ± 0.34		1 ± 0.26		0.89 ± 0.18	
	SCU	5.42 ± 1.79	1	3.76 ± 1.19	0.009	1.02 ± 0.21	0.99	0.87 ± 0.13	1
	SRP	5.44 ± 1.65		1.07 ± 0.08		0.87 ± 0.28		0.85 ± 0.01	
	CU	5.44 ± 1.76	1	1.39 ± 0.34	0.97	1 ± 0.26	0.99	0.89 ± 0.18	1
	SRP	5.44 ± 1.65		1.07 ± 0.08		0.87 ± 0.28		0.85 ± 0.01	

*P* value		1.000 †		≤0.001†		≤0.001†		≤0.001†	

SOD: superoxidase dismutase; C: control; EPD: untreated experimental diabetes and periodontitis; SCU: scaling and curcumin; CU: curcumin; SRP: scaling and root planing; †one-way ANOVA; *P*: probability <0.05 is significant; *P* > 0.05 is nonsignificant.

**Table 6:** The comparison between glucose levels in control and different groups using Dunnett *t* test and comparison between untreated EDP and three treated groups using Tukey HSD test, with one-way ANOVA† (*F*-test) to compare among different groups.

Variable	Groups	0 day		7 days		14 days		21 days	
		Mean ± SD	*P*	Mean ± SD	*P*	Mean ± SD	*P*	Mean ± SD	*P*
GLU (pg/dl)	C	113.96 ± 1.08	≤0.001	114.06 ± 0.98	≤0.001	114.14 ± 0.96	≤0.001	114.2 ± 1.11	≤0.001
	EDP	383.14 ± 1.99		388.86 ± 0.93		397.8 ± 5.07		406.62 ± 2.87	
	C	113.96 ± 1.08	≤0.001	114.06 ± 0.98	≤0.001	114.14 ± 0.96	≤0.001	114.2 ± 1.11	≤0.001
	SCU	382.72 ± 1.75		383.14 ± 1.99		383.14 ± 1.99		381.98 ± 2.31	
	C	113.96 ± 1.08	≤0.001	114.06 ± 0.98	≤0.001	114.14 ± 0.96	≤0.001	114.2 ± 1.11	≤0.001
	CU	382.62 ± 1.73		381.98 ± 1.71		384.8 ± 7.23		382.08 ± 3.37	
	C	113.96 ± 1.08	≤0.001	114.06 ± 0.98	≤0.001	114.14 ± 0.96	≤0.001	114.2 ± 1.11	≤0.001
	SRP	382.8 ± 1.69		379.74 ± 1.97		379.02 ± 1.24		378.26 ± 2.02	

*P* value		≤0.001†		≤0.001†		≤0.001†		≤0.001†	

GLU (pg/dl)	EDP	383.14 ± 1.99	0.98	388.86 ± 0.93	≤0.001	397.8 ± 5.07	0.001	406.62 ± 2.87	≤0.001
	SCU	382.72 ± 1.75		383.14 ± 1.99		383.14 ± 1.99		381.98 ± 2.31	
	EDP	383.14 ± 1.99	0.96	388.86 ± 0.93	≤0.001	397.8 ± 5.07	0.002	406.62 ± 2.87	≤0.001
	CU	382.62 ± 1.73		381.98 ± 1.71		384.8 ± 7.23		382.08 ± 3.37	
	EDP	383.14 ± 1.99	0.99	388.86 ± 0.93	≤0.001	397.8 ± 5.07	≤0.001	406.62 ± 2.87	≤0.001
	SRP	382.8 ± 1.69		379.74 ± 1.97		379.02 ± 1.24		378.26 ± 2.02	
	SCU	382.72 ± 1.75	1	383.14 ± 1.99	0.709	383.14 ± 1.99	0.93	381.98 ± 2.31	1
	CU	382.62 ± 1.73		381.98 ± 1.71		384.8 ± 7.23		382.08 ± 3.37	
	SCU	382.72 ± 1.75	1	383.14 ± 1.99	0.028	383.14 ± 1.99	0.50	381.98 ± 2.31	0.17
	SRP	382.8 ± 1.69		379.74 ± 1.97		379.02 ± 1.24		378.26 ± 2.02	
	CU	382.62 ± 1.73	0.999	381.98 ± 1.71	0.20	384.8 ± 7.23	0.22	382.08 ± 3.37	0.15
	SRP	382.8 ± 1.69		379.74 ± 1.97		379.02 ± 1.24		378.26 ± 2.02	

*P* value		0.970†		≤0.001†		≤0.001†		≤0.001†	

GLU: glucose; C: control; EPD: untreated experimental diabetes and periodontitis; SCU: scaling and curcumin; CU: curcumin; SRP: scaling and root planing; †one-way ANOVA; *P*: probability <0.05 is significant; *P* > 0.05 is nonsignificant.

**Table 7:** The comparison between body weight in control and different groups using Dunnett *t* test and comparison between untreated EDP and three treated groups using Tukey HSD test, with one-way ANOVA to compare among different groups.

Variable		0 day		7 days		14 days		21 days	
		Mean ± SD	*P*	Mean ± SD	*P*	Mean ± SD	*P*	Mean ± SD	*P*
BW (gr)	C	275.42 ± 3.38	0.17	278.9 ± 3.51	0.003	282.22 ± 3.18	≤0.001	289.74 ± 2.49	≤0.001
	EDP	268.84 ± 5.67		262.42 ± 7.18		257.08 ± 5.44		251.66 ± 2.65	
	C	275.42 ± 3.38	0.13	278.9 ± 3.51	0.004	282.22 ± 3.18	≤0.001	289.74 ± 2.49	≤0.001
	SCU	268.4 ± 5.58		263.04 ± 6.52		257.68 ± 5.31		253.12 ± 2.75	
	C	275.42 ± 3.38	0.12	278.9 ± 3.51	0.03	282.22 ± 3.18	≤0.001	289.74 ± 2.49	≤0.001
	CU	268.26 ± 5.48		267.16 ± 7.46		257.98 ± 5.2		254.28 ± 2.82	
	C	275.42 ± 3.38	0.12	278.9 ± 3.51	0.04	282.22 ± 3.18	≤0.001	289.74 ± 2.49	≤0.001
	SRP	268.28 ± 5.55		267.64 ± 7.13		258.14 ± 5.21		256.1 ± 1.32	

*P* value		0.167 †		0.005†		≤0.001†		≤0.001†	

BW (gr)	EDP	268.84 ± 5.67	0.99	262.42 ± 7.18	0.99	257.08 ± 5.44	0.99	251.66 ± 2.65	0.78
	SCU	268.4 ± 5.58		263.04 ± 6.52		257.68 ± 5.31		253.12 ± 2.75	
	EDP	268.84 ± 5.67	0.99	262.42 ± 7.18	0.71	257.08 ± 5.44	0.99	251.66 ± 2.65	0.36
	CU	268.26 ± 5.48		267.16 ± 7.46		257.98 ± 5.2		254.28 ± 2.82	
	EDP	268.84 ± 5.67	0.99	262.42 ± 7.18	0.65	257.08 ± 5.44	0.98	251.66 ± 2.65	0.05
	SRP	268.28 ± 5.55		267.64 ± 7.13		258.14 ± 5.21		256.1 ± 1.32	
	SCU	268.4 ± 5.58	1	263.04 ± 6.52	0.79	257.68 ± 5.31	1	253.12 ± 2.75	0.87
	CU	268.26 ± 5.48		267.16 ± 7.46		257.98 ± 5.2		254.28 ± 2.82	
	SCU	268.4 ± 5.58	1	263.04 ± 6.52	0.73	257.68 ± 5.31	0.99	253.12 ± 2.75	0.26
	SRP	268.28 ± 5.55		267.64 ± 7.13		258.14 ± 5.21		256.1 ± 1.32	
	CU	268.26 ± 5.48	1	267.16 ± 7.46	1	257.98 ± 5.2	1	254.28 ± 2.82	0.65
	SRP	268.28 ± 5.55		267.64 ± 7.13		258.14 ± 5.21		256.1 ± 1.32	

*P* value		0.998 †		0.546 †		0.989 †		0.067 †	

BW: body weight; C: control; EPD: untreated experimental diabetes and periodontitis; SCU: scaling and curcumin; CU: curcumin; SRP: scaling and root planing; †one-way ANOVA; *P*: probability <0.05 is significant; *P* > 0.05 is nonsignificant; *P* < 0.01 is highly significant.

## Data Availability

The data used to support the findings of this study are available from the corresponding author upon request.
